# Chronic pain among primary fentanyl users: The concept of self‐medication

**DOI:** 10.1002/ejp.4753

**Published:** 2024-11-06

**Authors:** Jane J. Kim, Dianah Hayati, Milad Zamany, Fiona Choi, Kerry Jang, Martha Ignaszewski, Pouya Azar, Michael Krausz

**Affiliations:** ^1^ Faculty of Medicine, Department of Psychiatry University of British Columbia Vancouver Canada; ^2^ Complex Pain and Addiction Service Vancouver General Hospital, DHCC Vancouver Canada; ^3^ BC Children's Hospital Vancouver British Columbia Canada

## Abstract

**Background:**

Chronic pain is among the leading causes of disability worldwide, of which only a small percentage of patients receive adequate treatment for. Non‐prescribed opioid analgesics are commonly sought out in effort to alleviate unrelieved pain. This study assesses the prevalence and correlates of chronic pain among primary fentanyl users.

**Methods:**

A cross‐sectional and structured survey was conducted with 200 adults who reported fentanyl as their drug of choice from a Vancouver acute care hospital. Presence and levels of chronic pain were determined through self‐report.

**Results:**

The majority of participants (*n* = 130, 72.6%) reported having chronic pain in the past 6 months, with the mean level of pain on a typical day to be 7.6 out of a scale of 10 (*SD* = 1.9). Majority (*n* = 85, 65.4%) reported using street opioids to self‐medicate, while only 9 (6.9%) reported that their chronic pain was unrelated. Regression analysis indicated that increasing age and co‐use of cannabis and opioids were independent associated factors of chronic pain. Higher levels of reported pain on a typical day were further associated with age and self‐medication.

**Conclusions:**

The findings of this study demonstrate a significant association between self‐medication and chronic pain among primary fentanyl users in British Columbia. For these individuals, inadequate pain relief may drive continued opioid use, which in turn may increase risks of treatment discontinuation and overdose. Appropriate pain management strategies are crucial to avoid opioid misuse and decrease the large societal burden caused by chronic pain.

**Significance:**

Our work points to the high prevalence of self‐reported chronic pain among individuals who primarily use fentanyl. Among those with self‐reported fentanyl use and chronic pain, self‐medication with street opioids was found to be common and associated with higher reported pain levels on a typical day. This highlights the need for pain management strategies to be integrated into opioid dependence treatment and more research in the overlap of pain and fentanyl use.

## 1 INTRODUCTION

Chronic pain is a common and complex problem, being the leading cause of global disability and disease burden (Vos et al., 2017). It is estimated to affect up to 1.9 billion people worldwide, and every one in five individuals in Canada (Schopflocher et al., 2011; Vos et al., 2017). Pain is an obstacle to the daily functioning and quality of life of those suffering (Dueñas et al., 2016). This is particularly relevant among people with opioid use disorder (OUD), who experience a high burden of chronic pain, with prevalence estimates varying from 48% to 60% (Voon et al., 2017).

The opioid overdose crisis is one of the most debilitating public health crises in Canada to date (Krausz et al., 2021). Between January 2016 and September 2023, there were a total of 42,494 apparent opioid toxicity deaths reported in Canada (Government of Canada, 2024). In British Columbia (B.C.), where some of the highest rates of unregulated drug‐related deaths were reported, fentanyl was detected in 85% of the unregulated drug deaths between 2023 and 2024 (BC Coroners Service, 2024). Fentanyl is a narcotic analgesic and synthetic opioid with both potency and rates of respiratory depression far more than that of heroin (Hill et al., 2020). Its arrival, followed by a quick proliferation, in the North American drug markets is a known driver of the surge in overdose events and deaths in recent years (Belzak & Halverson, 2018; Krausz et al., 2021). Consequently, efforts to address the crisis have focused on decreased access to opioid analgesics, withdrawing patients from their stable opioid regimens, and increased regulation of pain clinics (Bohnert et al., 2010; Fischer et al., 2016; Sw et al., 2016). Despite these efforts, the toll of the opioid crisis remains appallingly high, equating to about 6.2 B.C. deaths per day in March 2024 (BC Coroners Service, 2024).

Given the large proportion of opioid users who are affected by chronic pain, it is important to understand the degree of association between chronic pain and opioid misuse. Bicket et al. surveyed 203 people who inject drugs (PWID) in Maryland and found that almost half (47%) reported chronic pain, among which 76% used drugs to treat the pain, mostly with heroin (Bicket et al., 2020). While opioids are commonly sought after to manage pain, this association has yet to be investigated in the context of illicit fentanyl. The emergence of fentanyl has greatly impacted how people buy and use drugs as well as their perceived risk. Improving our understanding of chronic pain in those who intentionally use fentanyl may identify effective ways to treat their coexisting chronic pain and opioid dependence. To address these gaps, this paper examines the factors associated with self‐reported chronic pain among primary fentanyl users in Vancouver, British Columbia. We also examine the extent to which self‐reported pain severity is associated with street opioid use during a period of unprecedented crisis driven by fentanyl.

## 2 METHODS

The Fentanyl Cohort Study is a survey examining fentanyl as the primary drug of choice among patients of Vancouver General Hospital, a large acute care hospital in Vancouver, British Columbia. To be eligible, participants needed to be aged 18 or older, willing to provide informed consent, and report street opioid use in the past 6 months. Participants were recruited by convenience sampling from the hospital site and completed an interviewer‐administered questionnaire focusing on fentanyl use and treatment history. Survey items included the following: demographics, drug use characteristics, harm reduction behaviours, and health care utilization. All procedures for the study were approved by the Clinical Research Ethics Board of the University of British Columbia and Vancouver Coastal Health Security and Confidentiality Review Board. The study was funded by the Vancouver Coastal Health Research Institute Grant (F22‐01012). The present cross‐sectional analyses include data from the first 200 participants of the survey.

All participants were asked if they had experienced chronic pain (i.e. lasting for 3 months or longer) over the past 6 months. Once participants confirmed the presence of chronic pain, they were asked to rate their average pain level on a typical day on a scale ranging from 1 (very little pain) to 10 (worst possible pain). Participants also reported the location(s) of their pain from a list containing head/neck, chest/abdomen, back, hands/feet, arms, legs. They were given the option to verbally specify the location if elsewhere. Participants were asked how their opioid use was related to their chronic pain. Responses varied from self‐medication, losing access to prescription opioids (POs), POs not being strong enough, and having no access to POs (i.e. doctors will not prescribe them).

Bivariate analyses of all variables listed in Table 1 were conducted between participants identifying with and without chronic pain, with chi‐square tests for categorical variables and *t*‐tests for continuous variables. In a regression analysis, the presence of chronic pain was defined as the dependent variable and a forward‐fitting likelihood ratio procedure was used to add independent variables. Independent variables were identified as candidates for inclusion in the regression model if statistically significant in the bivariate analyses. Age and gender of participants were forced into the model. In those identifying with chronic pain, a separate linear regression model was constructed with the daily level of pain as the dependent variable and reported associations between opioid use and chronic pain as independent variables of interest. Statistical significance level for all analyses was set at *p* < 0.05, and all analyses were performed with SPSS version 29.

**TABLE 1:** Descriptive characteristics of participants identifying with and without chronic pain.

	Participants with chronic pain (*n* = 130)	Participants without chronic pain (*n* = 70)	*p*
Age			0.187
18–40	47 (36.2%)	32 (45.7%)	
41–77	83 (63.8%)	38 (54.3%)	
Gender			0.633
Male	81 (62.3%)	46 (65.7%)	
Female/Non‐binary	46 (65.7%)	24 (34.3%)	
Ethnicity			0.305
Indigenous	36 (27.7%)	17 (24.3%)	
Caucasian	77 (59.2%)	38 (54.3%)	
Other	17 (13.1%)	15 (21.4%)	
Highest level of education			0.133
Elementary to middle school	20 (15.4%)	9 (13.4%)	
Some high school	39 (30.0%)	15 (22.4%)	
High school certificate	25 (19.2%)	23 (34.3%)	
Post‐secondary	46 (35.4%)	20 (29.9%)	
Employment status over past 6 months			0.348
Employed	110 (85.9%)	50 (80.6%)	
Unemployed	18 (14.1%)	12 (19.4%)	
Self‐reported overdose occurrences in lifetime (mean, SD)	13.0 (33.3)	9.7 (18.7)	0.232*
Injected street opioids in past 6 months	23 (17.7%)	15 (21.4%)	0.521
Co‐used with street opioids in past 6 months			
Benzodiazepines	65 (50.0%)	25 (35.7%)	0.053
Stimulants	76 (58.5%)	42 (60.0%)	0.833
Alcohol	47 (36.2%)	18 (25.7%)	0.133
Cannabis	54 (41.5%)	19 (27.1%)	**0.044**
Use of street opioids everyday	49 (37.7%)	15 (21.4%)	**0.019**
Preference for fentanyl over other opioids	44 (35.5%)	24 (42.1%)	0.393
Actively looking to use fentanyl	49 (38.9%)	21 (35.6%)	0.667
Thinking of reducing or stopping fentanyl use	91 (70.0%)	33 (47.1%)	**0.001**
Previous participation in OAT	119 (91.5%)	53 (75.7%)	**0.002**
Currently receiving substance use disorder treatment	58 (44.6%)	21 (30.0%)	**0.044**
Diagnosed with mental health illness(es)	69 (53.1%)	29 (41.4%)	0.116
Visited hospital after an overdose	52 (54.7%)	21 (45.7%)	0.311
Would choose to visit a hospital after an overdose	50 (49.0%)	19 (37.3%)	0.218
Participated in medically supported detoxification program	74 (56.9%)	34 (48.6%)	0.258
Participated in rehab, bed‐based treatment or residential treatment in past 6 months	25 (19.2%)	14 (20.0%)	0.896

*Note*: *P* value obtained from chi‐square or t‐test (*); significant *P* values in bold.

## 3 RESULTS

A total of 179 participants were included in the analysis, with the majority (*n* = 130, 72.6%) reporting having suffered from chronic pain in the past 6 months. Demographic and clinical characteristics in participants with and without chronic pain are displayed in Table 1. Use of benzodiazepines (50.0% vs. 35.7%, *p* = 0.053) and cannabis (41.5% vs. 27.1%, *p* = 0.044) were significantly more common among individuals with chronic pain. At the time of interview, more individuals with chronic pain were likely to be thinking of reducing or stopping their fentanyl use (70.0% vs. 47.1%, *p* = 0.001) and to have histories of opioid agonist treatment (OAT) (91.5% vs. 75.7%, *p* = 0.002). However, the proportion of daily street opioid use was also significantly higher in those with chronic pain than without (37.7% vs. 21.4%, *p* = 0.019). No significant differences in the preference nor search for fentanyl were observed between the two groups.

**FIGURE 1:**
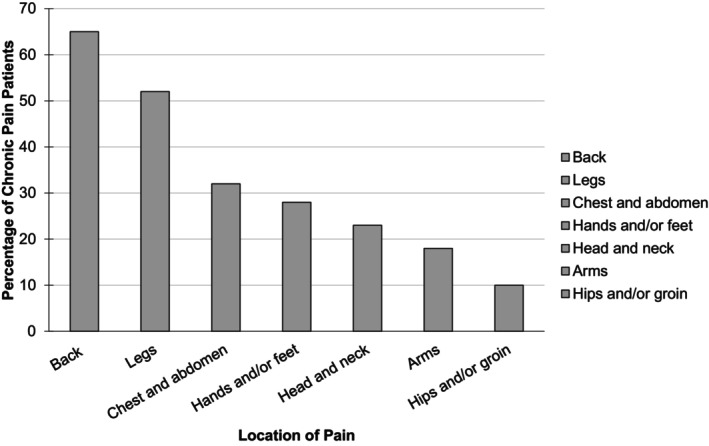
Pain locations in 130 participants reporting chronic pain over the past months. Multiple responses possible.

In participants with chronic pain, the mean level of pain experienced on a typical day was 7.6 (SD = 1.9) (Table 2). Majority (*n* = 85, 65.4%) reported using street opioids in an effort to self‐medicate. 25 (19.2%) reported that POs were not strong enough to alleviate their pain. Only 9 (6.9%) reported that their street opioid use was unrelated to their chronic pain. Figure 1 shows the locations of pain reported by participants. Participants often reported more than one location. More than half of the participants reported having back (65%) or leg (52%) pain, while close to a third reported pain in their chest or abdomen area (32%) or extremities (28%).

**TABLE 2:** Average levels of pain and self‐reported associations with street opioid use in participants identifying with chronic pain (*n* = 130). Multiple responses possible.

	*N* (%)
Average pain level on typical day (mean, SD)	7.6 (1.9)
1/10 (very little pain)	3 (2.3%)
2/10	4 (3.1%)
3/10	8 (6.2%)
4/10	14 (10.8%)
5/10	27 (20.8%)
6/10	33 (25.4%)
7/10	13 (10.0%)
8/10	28 (21.5%)
9/10	0 (0.0%)
10/10 (worst possible pain)	0 (0.0%)
Relationship of pain to street opioid use	
Self‐medication	85 (65.4%)
Lost access to prescription opioids	14 (10.8%)
Prescription opioids not strong enough	25 (19.2%)
Unable to get prescribed opioids	13 (10.0%)
Unrelated	9 (6.9%)

In analysis adjusted for age and gender, significant associations were found between age and cannabis use in the past 6 months with chronic pain (Table 3). Specifically, for each additional year in age, the odds of self‐reporting chronic pain were observed to increase by 4% (OR = 1.04, 95% CI = 1.01–1.08), and people who used cannabis in the past 6 months were found with 2.07 times higher odds of reporting chronic pain compared to those with no cannabis use (OR = 2.07, 95% CI = 1.02–4.20). Neither the want to reduce or stop fentanyl use or previous participation in OAT were found to be significantly associated with chronic pain.

**TABLE 3:** Factors independently associated with self‐reported chronic pain in primary fentanyl users.

	*p*‐value	OR (95% CI)
Age	**0.004**	1.044 (1.014–1.075)
Gender	0.417	0.761 (0.393–1.472)
Co‐use opioids with cannabis	0.043	2.074 (1.024–4.201)
Want to reduce or stop fentanyl use	0.074	1.859 (0.941–3.672)
Daily use of street opioids	0.077	1.859 (0.941–3.672)
Past participation in OAT	0.122	2.202 (0.811–5.979)
Currently receiving SUD treatment	0.409	1.334 (0.673–2.646)

*Note:* significant *P* values in bold.

Self‐reported associations between street opioid use and chronic pain were evaluated with pain levels experienced on a typical day (Table 4). Higher levels of reported pain on a typical day were significantly and positively associated with both age and self‐medication. For each additional year of age, there is an average increase of 0.281 units in pain experienced on a typical day (β = 0.281, *p* = 0.001). For those who self‐medicate compared to those who do not, there is an average increase of 0.265 units in pain experienced on a typical day (*β* = 0.265, *p* = 0.006).

**TABLE 4:** Self‐reported associations between street opioid use and chronic pain among primary fentanyl users identifying with chronic pain.

	*p*‐value	*β* (95% CI)
Age	**0.001**	0.281 (0.019–0.076)
Gender	0.189	−0.110 (−1.082–0.216)
Self‐medication	**0.006**	0.265 (0.318–1.813)
Lost access to prescription opioids	0.718	−0.031 (−1.223–0.845)
Prescription opioids not strong enough	0.101	0.145 (−0.140–1.547)
Unable to get prescribed opioids	0.122	0.136 (−0.233–1.962)
Unrelated	0.858	−0.016 (−1.474–1.229)

*Note*: significant *P* values in bold.

## 4 DISCUSSION

To our knowledge, this is the first study to examine associations between self‐reported chronic pain and street opioid use in a population of primary fentanyl users. Chronic pain was reported to be very common in this sample, affecting nearly three‐quarters in the past 6 months. Despite this considerable demand for pain care, participants described the lack of access to proper POs, whether not initiated, ineffective, or with limited access. The majority of those with reported chronic pain associated self‐medication with their street opioid use, and over a third reported using on a daily basis. Presence of self‐reported chronic pain was significantly associated with age and past 6‐month use of opioids with cannabis. Higher levels of pain experienced on a typical day were also significantly associated with age and self‐medication.

Our results support and extend previous research that chronic pain is associated with opioid misuse, driven by the need to cope with high levels of pain. Our sample consists entirely of individuals who are taking or have taken street versions of opioids in the past 6 months. The transition of obtaining opioids from medical settings to street buys has been cited as a negative harm of un‐ or undertreated pain. In one study, more than half (53.5%) of their sample turned to the unregulated supply once denied a legitimate prescription for pain (Piret et al., 2024). Another possibility is the transition from prescribed to illicitly produced opioids in those who were tapered rapidly or discontinued. In the 10 months after the Safe Prescribing of Opioids and Sedatives standard was released mid‐2016, switching to lower‐dose opioids among long‐term patients increased by an odds of 1.88 in B.C (95% CI 1.63 to 2.17) (College of Physicians and Surgeons of British Columbia, 2016; Morrow et al., 2019). Despite observed changes in the volume of opioids prescribed, physician adherence to guidelines raised new concerns of opioids tapered too quickly, leading patients with uncontrolled pain to seek alternative sources (Antoniou et al., 2019; Darnall et al., 2019; Dubin et al., 2017). Pain control, or the lack thereof, is an important consideration for treatment in people with opioid dependence. In our study, nearly all (91%) participants with chronic pain reported previous receipt of OAT, but less than half (45%) indicated current treatment. While retention in OAT has always been a significant challenge, limited evidence suggests even higher attrition rates in recent years, given fentanyl's propensity to worsen withdrawal and induce hyperalgesia (Mackay et al., 2021; McAdam et al., 2020). This suggests that uncontrolled pain may be a risk factor for poor adherence to OAT. While opioids are used to treat chronic pain, evidence for their long‐term effectiveness remains limited (Chou et al., 2015; Sehgal et al., 2013). Given the frequent occurrence of chronic pain in the opioid‐using population, it is possible that the loss of analgesic efficacy over time may result in patient dissatisfaction with the lower doses used in treatment and a turn toward illicit opioids with higher potency. Previous research has found an association between pain severity and illicit opioid use in those with comorbid chronic pain and OUD (Griffin et al., 2016). Our finding that most people with chronic pain (65.4%) associated self‐medication with their opioid use is similar to that found in other OUD patients, where chronic pain was reported by 43.2% as a primary factor in their relapse (Ellis et al., 2021). Careful attention to the recovery of both opioid dependence and pain is a requisite to patients to remain engaged in treatment as well as deterred from the illicit markets.

Participants from our sample were also twice as likely to report the co‐use of cannabis with opioids than those without chronic pain. In recent years, cannabis has received much attention in the field of pain management as both a substitute and adjunct for opioids. For example, cannabis use was associated with a 64% mean reduction in PO use among patients with chronic pain (Boehnke et al., 2016). High‐intensity use, defined as at least daily use in the past 6 months, was also associated with 21% greater odds of retention in OAT for patients who used illicit drugs (Socías et al., 2018). However, studies remain inconclusive on the consequences of their combined effects in the context of chronic pain. Compared to either opioid or cannabis use alone, the co‐use of both was found to be related to more anxiety, depression, and severity of opioid dependence among adults with chronic lower back pain (Buckner et al., 2023). This is in line with previous research demonstrating a joint association of cannabis and opioids with severe psychological distress among 12,358 adults in Ontario (Nigatu et al., 2022). The potential of concurrent cannabis‐opioid use to exacerbate mental health issues may interfere with the treatment of chronic pain (Cooke et al., 2019). With the widespread practice of polydrug use in recent years (Meyer et al., 2023), more research is needed to understand the interactive effects of opioids with cannabis. In our study, age was another factor associated with higher odds of having chronic pain. Pain has been found to generally increase with age, with older ages predicting both the onset of and failure to recover from chronic pain (Larsson et al., 2017). This makes older adults particularly vulnerable to opioid misuse and its consequences, including greater risk to injury and increased emergency department visits (Carter et al., 2019). More research is needed to better address the management of persistent pain among older adults.

It is of note that this sample consists entirely of individuals whose drug of choice is fentanyl. In North America, the time taken for fentanyl to dominate the drug markets is observed to have overlapped with when access to POs began to be constrained (Pardo et al., 2021). Following the rules of supply and demand, it is believed that the decline in hard‐to‐get POs was quickly supplanted with cheap‐to‐manufacture fentanyl (Krausz et al., 2021). In our sample, both those with and without chronic pain did not express a high preference for fentanyl nor sought it out actively. This shows that fentanyl may be an unwanted consequence of the current market distribution, given its high availability, low price, and popularity as a contaminant. Our analysis showed that self‐medication was significantly associated with higher reported levels of pain on a typical day (*β* = 0.265, *p* = 0.006). If moderate to severe pain is experienced over months to years, fentanyl may be the most cost‐effective method of procuring long‐term management. Others have also highlighted the critical role of financial sensibility in purchasing fentanyl, with its high potency outperforming the analgesic effects of heroin and other PO options being more “bang for your buck” (Urmanche et al., 2022). In addition, despite being active users at the time of interview, 70% of participants with chronic pain reported thinking of reducing or stopping the use of fentanyl. This result suggests that fentanyl use may be sustained by the need for pain relief. While the intention may be positive, the high risk of overdose that fentanyl carries remains a critical need to be met. As consumption preferences continue to shift to fentanyl (Ickowicz et al., 2022), further studies are needed to examine the role of chronic pain.

Several limitations of our study merit discussion. Our cross‐sectional design prevents causal inferences about whether unmanaged chronic pain leads to street opioid use, or if continued opioid use leads to chronic pain. Our sample was also drawn from a single hospital site in Vancouver, which may limit its generalizability. Additionally, the present analysis relied upon self‐reported data, which may have been subject to response bias and other errors. Although nearly three‐quarters (72.6%) of the sample reported suffering from chronic pain, it is possible that some of the included respondents would not meet criteria for a formal chronic pain diagnosis. Investigations based on objective measures of chronic pain would be useful to assess the validity of self‐reported chronic pain and its relationship to street opioid use.

Despite these limitations, our findings align with growing concerns of self‐medication in patients with inadequate pain relief. Importantly, the fact that a preference for nor actively seeking fentanyl were not predictors of chronic pain suggest that intentional use may be driven by the near universality of fentanyl in the current markets. Compounding this issue is the disproportionate access to therapeutic opioid dosages for those with lower incomes or belonging to racial and ethnic minority groups, which could be offsetting consumer preference and increasing propensity to purchase fentanyl for cheap (Meints et al., 2019). Given the growing cost of living, economic status is likely to reinforce or even worsen existing disparities in care and may compel those in pain to resort to the illicit market (Bardwell et al., 2021; Hansen et al., 2016). In addition, our study did not measure patient perceptions of available pain treatment. Future research could explore the specific domains of treatment that promote engaging patients with dual chronic pain and opioid use.

## 5 CONCLUSION

The surge in opioid‐related deaths worldwide calls not only for increased treatment options for opioid dependence, but also to improve the management of chronic pain as an underlying factor behind misuse and dropout. Though widely prevalent in opioid‐using populations, chronic pain is often overlooked in treatment. The reasons behind this are multifactorial and complex, including lack of clinician expertise, negative attitudes toward prescribing analgesics, and the labelling of patients as “drug‐seeking” (Dassieu et al., 2021). Our results highlight that chronic pain is a common phenomenon among people using fentanyl, and that the severity of pain is often addressed by self‐medication. Addressing the barrier of uncontrolled pain will be critical to reduce the risks of street opioid use as well as improve adherence to OAT. A multifaceted approach of maintenance therapy coupled with adjunctive pharmacological, psychological, and social treatment may be helpful to manage comorbid pain and decrease its large burden in this population (Koller et al., 2019; Wachholtz et al., 2011). As the proliferation of fentanyl continues, interventions that address patient needs and improve engagement are more critical than ever.

## AUTHOR CONTRIBUTIONS

This study was designed by M.I. and M.K. Data analysis was performed by J.K. and the results were examined by all authors. J.K. had a primary role in preparing the manuscript, which was edited by D.H., M.Z., F.C., K.J., P.A., and M.K. All authors have approved the final version of the manuscript.
